# Cognitive Training Sustainably Improves Executive Functioning in Middle-Aged Industry Workers Assessed by Task Switching: A Randomized Controlled ERP Study

**DOI:** 10.3389/fnhum.2017.00081

**Published:** 2017-02-22

**Authors:** Patrick D. Gajewski, Gabriele Freude, Michael Falkenstein

**Affiliations:** ^1^Ageing Research Group, Leibniz Research Centre for Working Environment and Human Factors (IfADo), Technical University of DortmundDortmund, Germany; ^2^Federal Institute for Occupational Safety and HealthBerlin, Germany; ^3^Institute for Working, Learning and AgingBochum, Germany

**Keywords:** aging, work, cognitive training, task switching, ERPs, N2, Ne, ERN

## Abstract

Recently, we reported results of a cross-sectional study investigating executive functions in dependence of aging and type of work. That study showed deficits in performance and electrophysiological activity in middle-aged workers with long-term repetitive and unchallenging work. Based on these findings, we conducted a longitudinal study that aimed at ameliorating these cognitive deficits by means of a trainer-guided cognitive training (CT) in 57 further middle-aged workers with repetitive type of work from the same factory. This study was designed as a randomized controlled trail with pre- (t1), post- (t2), and a 3-month follow-up (t3) measure. The waiting control group was trained between t2 and t3. The training lasted 3 months (20 sessions) and was evaluated with the same task switching paradigm used in the previous cross-sectional study. The CT improved performance in accuracy at the behavioral level and affected the electrophysiological correlates of retrieval of stimulus-response sets (P2), response selection (N2), and error detection (Ne), thus unveiling the neuronal background of the behavioral effects. The same training effects were observed in the waiting control group after CT at t3. Moreover, at t3, most of the behavioral and electrophysiological training-induced changes were found stable. Hence, CT appears to be an important intervention for compensating cognitive deficits in executive functions in middle-aged employees with cognitively unchallenging work.

## Introduction

The continuous aging of the society leads to an increasing average age of the workforce and an increase in the pensionable age at least in the majority of western countries (Ilmarinen, 2006). In addition, the working demands have changed in recent years: while physical work and physical demands have diminished successively, mental work and psychological work demands have increased (see Hagger et al., 2010, for an overview). Also, there has been an increasing trend for highly specialized work which in turn implies a larger amount of repetitive work which is done under increasing time pressure and worry about job loss due to globalization and cost pressure (Sparks et al., 2001; De Cuyper et al., 2008). This raises the problem whether and to which extent such modern working environments have a negative (or positive) impact on cognitive skills, and particularly so in older employees. Even more important is the issue of potential interventions that may ameliorate cognitive changes due to age and work and hence increase the workability of older employees.

It is well-known that extended cognitive engagement has a positive effect on cognition (Hultsch et al., 1999; Schooler et al., 1999; Wilson et al., 1999; Schooler and Mulatu, 2001; Singh-Manoux et al., 2003; Stern, 2009). Cognitively demanding work is an important cognitive stimulation supporting cognitive functions (Alvarado et al., 2002; Bosma et al., 2002; Wild-Wall et al., 2009; Marquié et al., 2010) which also lowers the risk of dementia in older age (Stern et al., 1994; Andel et al., 2005; Lee et al., 2010; Swaab and Bao, 2011).

In a longitudinal study, Schooler et al. (1999) investigated the effects of complex and cognitive demanding work on cognitive performance across 20 years. The authors showed that demanding jobs improved cognitive performance, particularly in older employees. Potter et al. (2008) observed a similar beneficial effect of demanding work in a sample of about 1000 older workers across a time range of 7 years, whereas tedious physical job demands tended to impair cognition in the workers regardless of age, intelligence, and education. Furthermore, in a recent longitudinal study, Marquié et al. (2010) analyzed performance in cognitive tasks as a function of cognitive work demands in about 3000 workers. The authors could show that the more cognitively stimulating the work the higher the scores in tests of episodic memory, attention and speed of processing. More importantly, cognitively stimulating work leads even to an increase in cognitive performance in the tested employees over 10 years despite their increasing age.

In the previous cross-sectional study (Gajewski et al., 2010b), we assessed cognitive functions of young and middle-aged blue-collar workers who have been working for a long time either under repetitive or under flexible working conditions. Both groups of middle-aged employees were matched regarding age- and education. In particular, we aimed at investigating the status of cognitive control functions involved in a memory-based task-switching task, namely task preparation, working memory, and error processing. These functions were investigated by analyzing a specific ERP-component which reflects the cortical activity underlying different aspects of processing during task switching. We observed no age- and work-related impairments when the relevant task was indicated by a cue which reduced the working memory load. Age differences were apparent when memory demands were higher, i.e., when the task sequence had to be maintained across a large number of trials (memory-based task switching). Importantly, the older workers with long-term repetitive work showed the strongest deficits in performance. These behavioral impairments were accompanied by specific findings in the ERP, namely attenuation of preparation, reduction of working memory capacity, and diminished error detection. In contrast, the older workers with long-term flexible work showed small or no performance and ERP detriments when compared to young workers but differed considerably from their contemporary, repetitive working colleagues. These results are in line with the literature reporting negative impact of long-term unchallenging work on cognition.

Nonetheless, since our previous study was a cross-sectional one, the impairment of cognition in the workers with long-term repetitive work could also be due to early selection mechanisms, i.e., workers with relatively low cognitive skills prefer work in such repetitive jobs. While this is unlikely, given that the deficit was only seen in the demanding memory-based switching condition, it cannot be excluded. Therefore, a longitudinal study would be necessary to control for possible confounding variables and to analyze the time-course of cognitive functions across decades in workers with different working conditions. More useful, however, would be a longitudinal study which examines methods to improve cognitive functions (Stern, 2009; Gajewski and Falkenstein, 2015b). More specifically, it should be analyzed whether the cognitive deficits and their electrophysiological correlates found in our previous study could be ameliorated by cognitive training (CT).

Indeed, in the literature the most often reported type of formal CT is training of a specific function that focuses on one domain only, for example memory (Jaeggi et al., 2008), attention (Green and Bavelier, 2008), visual search (Becic et al., 2008), dual task (Bherer et al., 2005), or task switching (Karbach and Kray, 2009). In most studies the training effects were restricted to the trained function and did not transfer to other functions (e.g., Willis and Schaie, 1986; Willis et al., 2006), while some reports showed also transfer effects on non-trained activities (e.g., Gopher et al., 1994; Ball et al., 2007; Caserta et al., 2007; Cassavaugh and Kramer, 2009; Edwards et al., 2009; Karbach and Kray, 2009). Yet, the trained function could be influenced by an implicit involvement of non-explicitly trained functions.

As a consequence from the training literature it appears beneficial to conduct training that involves several fluid functions relevant for work and daily life (Kramer and Morrow, 2008). In most CT studies, test-like tasks or games which target different cognitive functions were trained via PC (computerized cognitive training; CCT) for an extended time. Recent meta-analyses suggest that CCT leads to improvements in various cognitive functions and also transfers to untrained cognitive tasks or even everyday situations (far transfer) in healthy older adults (Karbach and Verhaeghen, 2014; Kelly et al., 2014; Lampit et al., 2014; Ballesteros et al., 2015; Gajewski and Falkenstein, 2015b).

### ERPs in the Task Switching Paradigm

Studies evaluating training-related effects using electrophysiological measures are scarce despite the advantage of this method to investigate distinct processing steps from stimulus analysis across memory activation, decision making to response-related processes. In the present study, we focus primarily on cognitive processes associated with particular ERP components: retrieval of stimulus-response sets (P2), response selection (N2), allocation of cognitive resources to the task (P3b) and response-related processes like response monitoring (Nc) and error detection (Ne). The P2 is a frontocentral positive wave with latency of about 170–200 ms that has been associated with evaluation of task relevant stimuli and retrieval of S-R mappings (Kieffaber and Hetrick, 2005; Gajewski et al., 2008; Adrover-Roig and Barceló, 2010). Larger P2 amplitudes may reflect enhanced target processing under difficult conditions such as task switch trials where larger P2 amplitudes were related to slower responses (Finke et al., 2011). Moreover, P2 latency was positively correlated with reaction times (RTs), supporting the interpretation of P2 as an index of S-R retrieval (Kieffaber and Hetrick, 2005).

Following the P2 the frontocentral negativity, the N2, with a latency of about 280–320 ms occurs. The N2 was mainly associated with mismatch (Folstein and Van Petten, 2008, for review), cognitive control of response (Hämmerer et al., 2014), conflict processing (Van Veen and Carter, 2002; Yeung and Cohen, 2006), decision making (Ritter et al., 1979), and response selection (Gajewski et al., 2008). In task switch trials interference processing between conflicting task-sets (i.e., S-R sets) was often associated with larger N2 amplitude and longer latency than in non-switch trials (Gajewski et al., 2010a, 2011; Karayanidis et al., 2011). This process seems to be sensitive to training as the N2 increased substantially after 4 months of CT in seniors (Gajewski and Falkenstein, 2012).

The P3b is a large positive wave with parietal maximum peaking at about 300 to 600 ms after stimulus onset that is associated with context updating (Donchin and Coles, 1988), allocation of cognitive resources (Kok, 2001), working memory (Polich, 2007), and outcome of a decision process (Nieuwenhuis et al., 2005; Verleger et al., 2006). The P3b was frequently reported in task switching studies with larger amplitudes in non-switch than task switch trials (e.g., Barceló et al., 2000; Lorist et al., 2000; Rushworth et al., 2002; Jost et al., 2008; Gajewski and Falkenstein, 2011). Training effects on the P3b are inconsistent. However, generally larger P3b amplitude was associated with better performance (Polich and Lardon, 1997; Hillman et al., 2006).

During response execution an error may occur. Such response errors are usually followed by a negative wave with a frontocentral maximum, the error negativity (Ne; Falkenstein et al., 1991), or error-related negativity (ERN; Gehring et al., 1993) which is thought to reflect the detection of errors or incorrect response tendencies. The Ne is seen as the result of a comparison process between the expected and the actual outcome, leading to behavioral readjustment and reflects a precondition for successful learning (Falkenstein et al., 2000; Holroyd and Coles, 2002). It has also been shown that the Ne is smaller for older than younger participants, suggesting a weakening of action monitoring processes in older adults (Band and Kok, 2000; Falkenstein et al., 2001). After correct responses, a smaller negativity is seen, called Nc or CRN (Yordanova et al., 2004) which is thought to reflect response monitoring (Allain et al., 2004).

### The Present Study

The aim of the longitudinal part of the cross-sectional study reported by Gajewski et al. (2010b) was to ameliorate cognitive decline found in middle-aged assembly line workers, as outlined above. In the present study, CT as a crucial method for improving fluid cognition as reported in the literature (Strobach and Karbach, 2016) was applied. We used a formal paper- and computer-based CT which was found to be effective in improving cognitive functions in healthy older subjects as measured with behavior as well as electrical brain activity in the previous training study (Gajewski and Falkenstein, 2012; Wild-Wall et al., 2012). The training was not focused on a particular cognitive function but aimed at affecting a broad spectrum of cognitive abilities with different training tasks. The CT contained both several paper and pencil tasks (Mental Activation Training, MAT; Lehrl et al., 1994) and a number of computer-based tasks from commercial packages. The tasks were selected to target different cognitive functions such as attention, working memory, inhibition, and dual-task performance. The training format was two sessions per week (lasting 1.5 h each) for 3 months in groups guided by a skilled teacher. Fifty-seven middle-aged workers with highly repetitive work finished this study. The participants were randomly assigned to the training group and a Waiting Control Group. The latter received a combined cognitive, relaxation, and stress management training after the Cognitive Training Group finished the training, i.e., after both groups were post-measured. The offer of the combined training was aimed to keep the motivation of the waiting group high and to reduce dropouts and also to assess effects of the stress management training (which will be reported elsewhere). Moreover, this design allows additional validation of CT effects in a second sample. Apart from behavioral data we aimed at analyzing a number of ERPs which may be affected by CT.

Following hypotheses were formulated: Existing evidence indicates that CT is a useful tool for enhancing cognitive functions (Strobach and Karbach, 2016). Thus, CT should be particularly helpful in restoring cognitive deficits in individuals with long-term repetitive and unchallenging type of work. To measure the effects of CT, the same difficult memory-based task switching paradigm was used as in the previous study that revealed cognitive impairments in assembly line workers (Gajewski et al., 2010b). This task should be suitable to detect even marginal training-induced changes in executive functioning in middle-aged healthy workers. According to the findings of our previous training study with healthy seniors (Gajewski and Falkenstein, 2012), we expected improvement of accuracy in this task, whereas no changes in speed were expected. Moreover, performance effects should be accompanied by specific enhancements in ERP activity. Specifically, we expect an enlargement of N2 and P3b amplitudes after the training, indicating more efficient response selection, and working memory processes, as well as increased Ne/ERN, suggesting improved error detection that would corroborate our previous results. In respect to the P2, we also expect larger amplitudes after CT as enhanced performance was associated with larger P2 amplitudes (Wild-Wall et al., 2012). We do not have any specific expectations regarding the long-term effect of training assessed by the follow-up measure. Finally, we do not expect any beneficial effects of a combined relaxation and CT over a pure CT on executive functions, as relaxation alone was not associated with cognitive effects (Gajewski and Falkenstein, 2012).

## Materials and Methods

### Participants

From the 60 participants 57 healthy male volunteers aged from 40 to 57 (*M* = 46.5, *SD* = 4.5) finished the study and completed all measures. None of the individuals participated in the cross-sectional study conducted previously (Gajewski et al., 2010b). Eleven participants were left- or both handed. They had normal or corrected-to-normal vision. Five participants finished Primary school (4th grade), 34 secondary general school (10th grade), 13 intermediate secondary school (10th grade), and 5 grammar school (gymnasium, 12th grade). Twenty-eight participants worked in double shifts (early and late shift), 20 participants worked in a night shifts only. Early and night shift employees were trained together in the afternoon after the early shift was finished. Late shift employees were trained before the late shift began. All participants received a payment for their participation. This study was carried out in accordance with the recommendations of the local ethics committee of the Leibniz association with written informed consent from all subjects. All subjects gave written informed consent in accordance with the Declaration of Helsinki. The protocol was approved by the local ethics committee of the Leibniz association.

### Study Design

**FIGURE 1:**
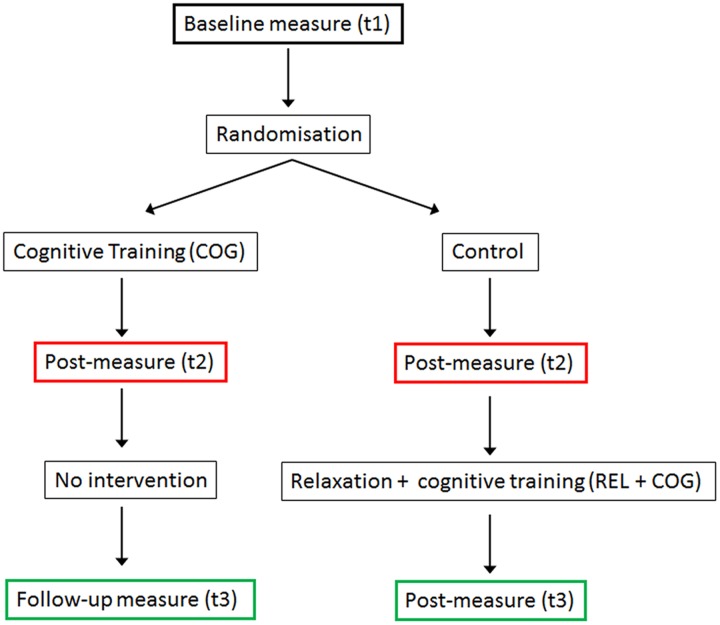
**A scheme of the study design**.

The schedule of trainings and measurements is presented in **Figure 1**. The study was designed as a randomized controlled trial with pre-, post-measure, and a follow-up measures. After the pre-measure (t1), participants were randomly assigned to the cognitive training (COG) and a Waiting Control Group. After the 20 sessions of the CT were finished (t2) both groups were tested again. Thereafter the Control Group received a stress management and relaxation training (REL) in the first eight sessions and in the remaining 12 sessions qualitatively the same (though shorter) cognitive training (REL + COG). After the combined training was finished both groups were measured again (t3), providing a follow-up measure for the COG group and an assessment of the training changes in the combined training group (REL + COG). Note, though the main focus was on the effects of the COG-group relative to the control between t1 and t2 and the long-term effect of training assessed at t3, we also report the effects of the combined intervention to investigate whether the training effects were similar to that for the COG group. Before, after the training and 3 months after the end of the training several paper and pencil and computer-based psychometric tests were applied. Here, we report only the results of the PC-based task switching paradigm.

### Cognitive Training

The CT lasted 3 months with two sessions per week and 1.5 h per session (20 sessions in total). The training aimed at enhancing general cognitive abilities and was not designed to improve a particular cognitive function. Thus, the training consisted of a paper and pencil and a number of commercial and non-commercial PC-based exercises. A number of training packages listed below were already used and described in a previous study (cf. appendix in Gajewski and Falkenstein, 2012).

During the first month the so-called MAT (Lehrl et al., 1994) was applied. MAT is a paper-based CT to increase basic cognitive functions such as short-term memory and speed of information processing. In the subsequent weeks, the participants were trained with selected tasks taken from commercial and non-commercial internet-based software packages^1^^,^^2^^,^^3^^,^^4^^,^^5^. The difficulty level of the training tasks was continuously adapted to the participants’ individual abilities either by the program or by the trainer.

Each session comprised different exercises that aimed to train crucial cognitive functions like perceptual speed, attention, and memory. For example, the training package FreshMinder2 includes games that require fast responding to specific stimuli like colored balloons, fast selecting digits in ascending order, memorizing and delayed recalling of faces, repeating of sound sequences, matching of letters, memorizing shopping lists, counting bricks in 3-D figures, memorizing and recalling of schematic paths, etc.

The package Ahano peds consists of units with different difficulty levels. The freely available program includes an eye–hand coordination task, money counting task, detection of word repetitions in a text, block tapping task, memorizing abstract figures etc. The package Mentaga consists of exercises enhancing vigilance, perceptual speed, spatial cognition, and eye–hand coordination. Finally, the package Mental-Aktiv offers a number of memory tasks using digits, letters, colors and figures and exercises to train speed of processing. The specific tasks from those packages were chosen on the basis of their validity for the targeted functions as well as the fun they induced during exercising.

Additional sessions took place at the end of the program for those participants who missed the regular sessions.

### Task Switch Task

The same task was used as in a number of previous reports (Gajewski et al., 2010b; Gajewski and Falkenstein, 2012, 2015a; Schapkin et al., 2014). Briefly, the stimuli in the task switching paradigm consisted of the digits 1–9, excluding the number 5. The digits were presented in white on a black computer screen 3 mm above the white fixation point (10 mm diameter). Each digit was presented in small (7 mm × 10 mm) and in large (12 mm × 18 mm) size. A cue (16 mm × 32 mm) indicating the relevant task was presented 3 mm below the fixation point. The cue “NUM” (German “Numerisch,” numeric) indicated a numerical task (greater or less than 5), “GER” (German “Geradzahligkeit,” parity) the parity task (odd vs. even), “SCH” (German “Schrift,” font) the font-size task (small vs. large). The cue was presented in the single tasks only. In the memory-based mixed block (see below) three letters X were presented instead of the informative cue. Responses consisted of pressing one of two buttons which were mounted in a response box. The buttons should be pressed with the index fingers. The stimulus–response mapping of the three tasks was overlapping, that is, responses according to ‘smaller than five,’ ‘even,’ and ‘small size’ were assigned to the left key and ‘larger than five,’ ‘odd,’ and ‘large size’ to the right key. This assignment was counterbalanced across participants.

### Procedure

**FIGURE 2:**
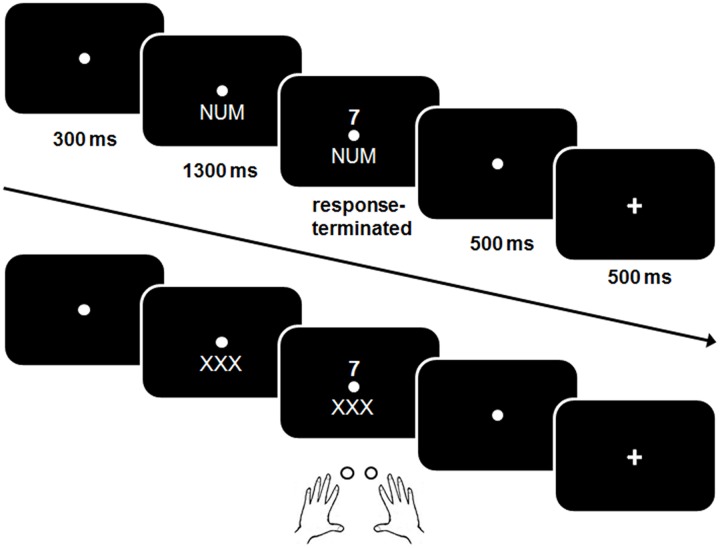
**Schematic illustration of a trial in the single (practice) block and a mixed task block (bottom)**. In contrast to the single block, no explicit cue was presented in the mixed block.

A schematic example of a trial is shown in **Figure 2**.

The same procedure was used as in the previous studies that used this task (Gajewski et al., 2010b; Gajewski and Falkenstein, 2012, 2015a; Schapkin et al., 2014). Shortly, a trial started with a presentation of the fixation point for 300 ms. In the single task blocks, a cue stimulus was presented for 1300 ms which remained visible when the digit was presented. A response had to be given within 2500 ms after target-onset. Five hundred milliseconds after the response a feedback was displayed for 500 ms. In case of a correct response a plus sign, after a false response a minus sign was displayed. After that the next cue was shown. The response-cue interval (RCI) was set to 1000 ms and included the response-feedback delay and the feedback.

At the beginning of the session participants performed three single task blocks with a fixed task NUM, GER, and SCH consisting of 34 trials each. The single task blocks were used to become familiar with the different task rules before the mixed block was run and to assess baseline performance in a task without a concurrent task.

In the memory-based mixed block, the three tasks were presented in mixed order and the participants were instructed to switch the task after every three trials in the following order “NUM–NUM–NUM–GER–GER–GER–SCH–SCH–SCH” etc. while a dummy cue “XXX” instead of a cue was presented, i.e., participants had to keep the trial sequence in mind. When three consecutive errors were made or no response within the 2500 ms interval was given, cues were presented for the next three trials, helping the participants to find the track. The mixed block consisted of 126 trails.

The frequency of task switch in the memory-based block amounted to 33.3% of trials. The participants were given written instructions explaining the task. The instructions encouraged quick and accurate responses.

### EEG Recording

The same recording parameters were used as in our previous studies (Gajewski et al., 2010b; Gajewski and Falkenstein, 2012, 2015a). Briefly, EEG was recorded from 32 scalp electrodes according to the extended 10–20 system and mounted on an elastic cap. The montage included 8 midline sites and 12 sites on each hemisphere and two mastoid electrodes (M1 and M2). The EEG was re-referenced offline to linked mastoids. The horizontal and vertical EOG was recorded bipolarly from electrodes at both eyes. Eye movement artifacts were corrected using the correction algorithm of Gratton et al. (1983). Electrode impedance was kept below 10 kΩ. The amplifier bandpass was 0.01–140 Hz. EEG and EOG were sampled continuously with a rate of 2048 Hz. Offline, the EEG was downscaled to a sampling rate of 1000 Hz and cut in stimulus-locked by using the software Vision Analyzer (Brain Products, Munich). Epochs in which the amplitude exceeded ±150 μV were rejected. The ERPs were filtered digitally offline with a 17 Hz low and 0.05 Hz high pass.

### Data Analysis

Excluded from the RT analysis were: the first trial of each test block, trials with responses faster than 100 ms or slower than 2500 ms and error trials. RTs and error rates (ERs) were subjected to an ANOVA design including within-subject factors Task-Set Transition (non-switch vs. switch), Session (pre-test (t1) vs. post-test (t2)) and the between-subject factor Group (training vs. control).

As the design was not fully balanced (CT between t1 and t2 and combined training between t2 and t3, see **Figure 1**) the follow-up effect in the COG was assessed by planned comparisons t2 vs. t3 and t1 vs. t3 within this group. ANOVAs were conducted with the two within-subject factors Task-Set Transition and Session without the between subject factor Group. A significant effect of Session between t1 and t3 and lack of difference between t2 and t3 would suggest a training-induced change that persists until the follow-up measure.

The training effect in the combined training group (REL + COG) was assessed by planned comparisons between t2 and t3 and t1 vs. t2. Similar to the planned comparisons used in the COG group, ANOVAs with the two within-subject factors Task-Set Transition and Session within this group were conducted. A substantial training effect would be found when a change occurs between t2 and t3 whereas no difference will be found between t1 and t2 (effect of repeated measures).

Behavioral and ERP data were analyzed from the memory-based mixing block. The length of the epoch in the target-locked ERP was 1100 ms, and 600 ms in the response-locked ERP. The ERP analysis was restricted to the post-target and post-response ERPs at the midline electrodes (Cz and Pz) as the targeted components P2, N2, P3b, and Nc/Ne are usually maximum at the midline. Moreover, *a priori* selection of these electrodes considerably reduced the complexity of the reported effects. In contrast to our previous studies, we analyzed the P2 at Cz and not at Fz as the P2 amplitude was clearly maximum at Cz. The P2 was measured as the most positive peak between 150 and 300 ms. N2, P3b, and Ne were analyzed at the same electrodes as in the previous reports (Gajewski et al., 2010b; Gajewski and Falkenstein, 2012, 2015a). The N2 was measured as the most negative peak at Cz in the time range 150–400 ms after target-onset. The P3b was measured at Pz in the time range 300–600 ms after target-onset. P2, N2, and P3b were measured in correct trials relative to 100 ms baseline prior to target – onset. Nc and Ne were measured in as the most negative peak in the time range 0–150 ms after the response relative to the 100 ms pre-response baseline. Nc and Ne were collapsed across non-switch and switch trials to get at least six error trials per subject (Steele et al., 2016). The ERPs were analyzed in the same way as the behavioral data.

## Results

### Behavioral Data

**FIGURE 3:**
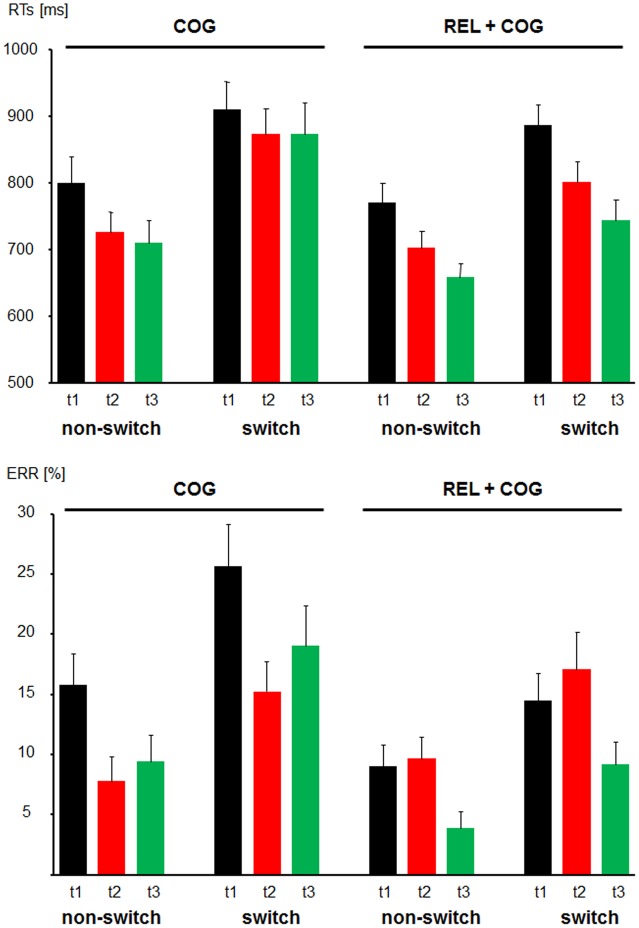
**Behavioral data**. Reaction times **(Top)** and error rates **(Bottom)** as a function of Task-Set Transition (non-switch and switch) in the Cognitive Training Group (between t1 and t2; COG) and Waiting Control Group (REL + COG). T3 in the COG group reflects the follow-up measure, whereas t3 in the REL + COG reflects effect of the combined relaxation and cognitive training.

Mean RTs and ERs for the non-switch and switch trials for the training and the Control Group are presented in **Figure 3**.

For the analysis of response times, error trials (8.5 and 6.0%) and outliers (0.9 and 0.4%) for the training and Control Group, respectively, were discarded.

### Reaction Times

For the group comparison between t1 and t2 the mean RTs in non-switch trials were shorter than in switch trials (750 vs. 868 ms), reflecting substantial switch costs [*F*(1,56) = 66.8, *p* < 0.0001, η^2^ = 0.544]. The factor Session was significant due to faster RTs in the post- than pre-measure [776 vs. 846 ms; *F*(1,56) = 8.8, *P* < 0.005, η^2^ = 0.137]. None of these factors were modulated by the factor Group.

The follow-up effect in the COG group assessed by the comparison between the Sessions t2 and t3 confirmed again an effect of Task-Set transition [*F*(1,25) = 39.1, *p* < 0.0001, η^2^ = 0.610] but no effect of Session nor interaction between Task-Set Transition and time were significant (both *F*’s < 1). The comparison between t3 and t1 showed, apart from the effect of Task-Set Transition [*F*(1,25) = 24.2, *p* < 0.0001, η^2^ = 0.493], only trends for the effect of Session [*F*(1,25) = 2.7, *p* = 0.114, η^2^ = 0.097] and the interaction of both factors [*F*(1,25) = 2.5, *p* = 0.123, η^2^ = 0.092], suggesting no stable change of RTs due to training.

In order to assess a possible training effect in the REL + COG group, t2 was compared with t3. ANOVA yielded an effect of Task-Set Transition [*F*(1,30) = 40.8, *p* < 0.0001, η^2^ = 0.557], which was not modulated by time-point (*F* < 1). However, there was a trend of Session, suggesting faster RTs at t3 than at t2 in this group [701 vs. 740 ms, *F*(1,30) = 3.4, *p* = 0.076, η^2^ = 0.101]. For the comparison t2 vs. t1 the effect of Task-Set Transition was again significant [*F*(1,30) = 62.3, *p* < 0.0001, η^2^ = 0.675] as well as the effect of Session [*F*(1,30) = 11.0, *p* < 0.005, η^2^ = 0.269]. The interaction did not reach significance [*F*(1,30) = 1.4, *p* = 0.245, η^2^ = 0.045]. Basically, no changes in speed due to training were observed. RT reductions were merely due to repeated measures.

### Error Rates

The main effect of Task-Set Transition was significant [*F*(1,56) = 76.5, *p* < 0.0001, η^2^ = 0.577], suggesting switch costs in accuracy (10.5 vs. 18.1%, for non-switch and switch trials, respectively). The ER was lower at t2 than t1 resulting in the effect of Session [12.4 vs. 16.2%; *F*(1,56) = 5.9, *p* < 0.05, η^2^ = 0.096]. Importantly, the factor Session was modulated by Group [*F*(1,56) = 12.2, *p* < 0.001, η^2^ = 0.179] due to a reduction of ERs from t1 to t2 in the COG group from (20.7 to 11.5%) and a slight increase in the Control Group (11.7 vs. 13.4%).

The follow-up analysis in the COG group revealed an effect of Task-Set Transition [*F*(1,25) = 49.8, *p* < 0.0001, η^2^ = 0.666] and no effect of Session or interaction of both factors (both *F*’s < 1). Comparison between t3 and t1 yielded an effect of Task-Set Transition [*F*(1,25) = 36.7, *p* < 0.0001, η^2^ = 0.595] and an effect of Session [*F*(1,25) = 4.7, *p* < 0.05, η^2^ = 0.159], whereas no interaction between Task-Set Transition and Session was found (*F* < 1).

After the training between t2 and t3 the REL + COG group showed apart from the expected effect of Task-Set Transition [*F*(1,30) = 36.5, *p* < 0.0001, η^2^ = 0.549] a main effect of Session [*F*(1,30) = 10.7, *p* < 0.005, η^2^ = 0.264], whereas the interaction Session × Task-Set Transition did not reach significance [*F*(1,30) = 1.4, *p* = 0.236, η^2^ = 0.049]. The comparison t1 vs. t2 did not reveal effect of Session or interaction Session × Task-Set Transition and showed merely the effect of Task-Set Transition [*F*(1,30) = 33.2, *p* < 0.0001, η^2^ = 0.518].

In order to analyze which participants benefitted from the CT we split the COG group in low and high performers (i.e., lower vs. higher ERs at t1 in task switch condition than the median). The analysis revealed an interaction Session × Performance [*F*(1,24) = 10.1, *p* < 0.005, η^2^ = 0.297], and a second order interaction Session × Task-Set Transition × Performance [*F*(1,24) = 13.8, *p* < 0.001, η^2^ = 0.365], suggesting that the low performers improved the accuracy after training particularly in task switch trials between t1 and t2 (41.2 vs. 20.1%), whereas high performers did not (10.0 vs. 10.4%) improve. The benefit was less pronounced in non-switch trials (23.6 vs. 12.9% in the low performers and 8 vs. 3.3% in the high performers). Moreover, age was differentially associated with training gains. Younger trainees (40–47 years) benefitted strongly from the training (14% accuracy improvement) than their older colleagues (47–57 years, 2% improvement), resulting in an interaction Age Group × Session [*F*(1,24) = 5.5, *p* < 0.05, η^2^ = 0.187].

In sum, the ERs were reduced in the COG group after training. This reduction remained unchanged during the follow-up measure. In the Waiting Control Group, no changes were found between t1 and t2 but again a clear reduction of ERs was observed after the combined training at t3. No specific changes of local switch effects in accuracy due to training were seen. Initial low performers benefitted more from the training than high performers, particularly in task switching trials. Younger trainees improved their accuracy after training more than the older ones.

### ERP Data

**FIGURE 4:**
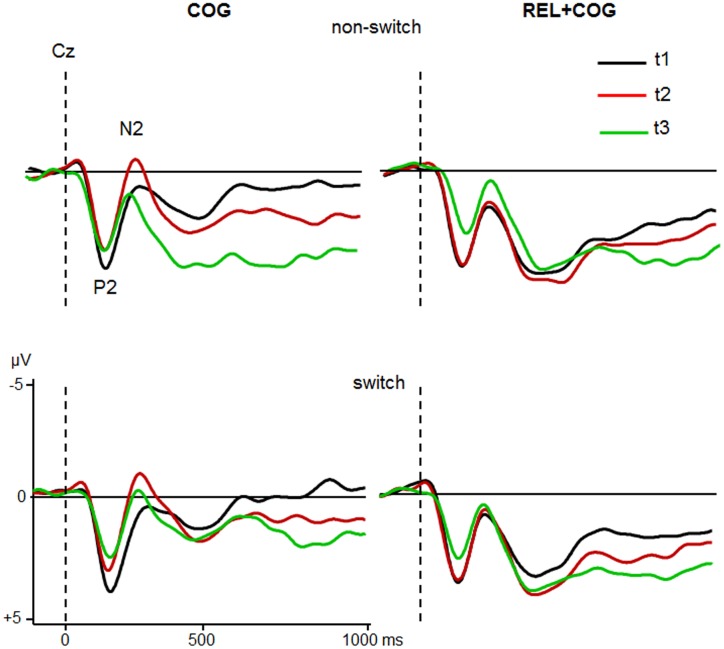
**ERPs at Cz of the COG and the Waiting Control Group (REL + COG) as a function of Task-Set transition for the Sessions t1 (black), t2 (red), and t3 (green)**. See caption of **Figure 3** and the study design for more information.

**FIGURE 5:**
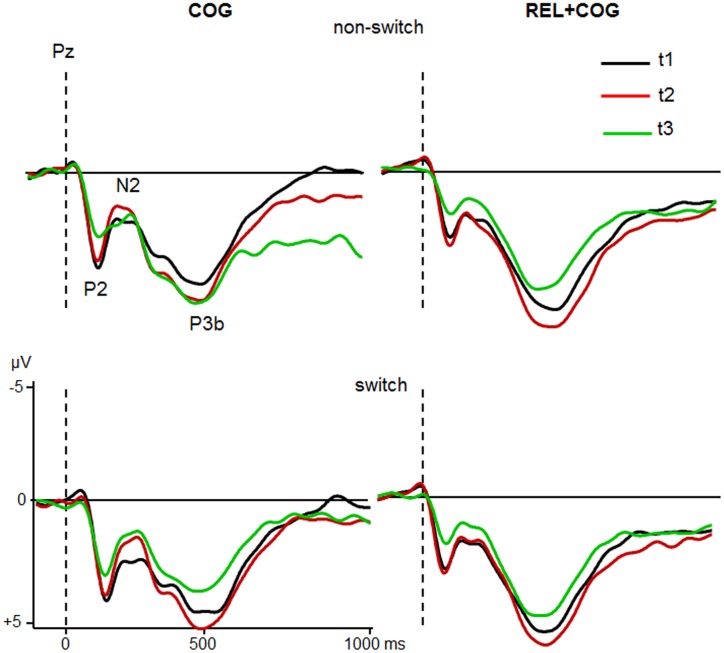
**ERPs at Pz of the COG and the Waiting Control Group (REL + COG) as a function of Task-Set transition for the Sessions t1 (black), t2 (red), and t3 (green)**. See caption of **Figure 3** and the study design for more information.

Grand averages for target-locked ERP-waveforms at Cz and Pz for the two groups are shown in **Figures 4** and **5**.

In order to focus on training-related changes of executive functions, only effects or interactions including Task-Set Transition, Session, and Group were reported in the following.

### ERPs

#### P2

The ANOVA analyzing effect of the COG training relative to the Waiting Control Group revealed a trend for an interaction Session × Group for P2 latency [*F*(1,56) = 4.2, *p* < 0.05, η^2^ = 0.069]. This interaction was due to reduced P2 latency at t2 relative to t1 in the COG group (158 vs. 167 ms), whereas no such effect was observed in the Control Group (161 vs. 158 ms). Also, there was a trend for the interaction Session × Group × Task-Set Transition [*F*(1,56) = 3.2, *p* = 0.078, η^2^ = 0.055], suggesting that the latency reduction was mainly due to task switch (14 ms) and hardly in non-switch trials (5 ms). P2 amplitude showed an effect of Session [*F*(1,56) = 10.7, *p* < 0.005, η^2^ = 0.161], indicating a general reduction of P2 at t2 compared to t1 (5.3 vs. 4.7 μV) that was additionally modulated by the factor Group [*F*(1,56) = 9.6, *p* < 0.005, η^2^ = 0.147]. This interaction was based on the amplitude reduction between t1 and t2 in the COG group (5.4 vs. 4.2 μV) while no changes in the Waiting Control Group were observed (5.1 vs. 5.1 μV). The assessment of the follow-up effect for P2 latency and amplitude (t2 vs. t3) yielded no effect of Session nor interaction Session × Task-Set Transition (all *F*’s < 1). However, the comparison between t1 and t3 revealed a substantial effect of Session for P2 amplitude (5.4 vs. 3.9 μV; *F*(1,23) = 19.0, *p* < 0.0001, η^2^ = 0.354) but not for P2 latency (*F* < 1).

Comparison between the Sessions t2 and t3 for the Waiting Control Group (REL + COG) showed for the P2 latency a main effect of Task-Set Transition due to delayed P2 in non-switch than switch trials [168 vs. 159 ms; *F*(1,30) = 6.0, *p* < 0.05, η^2^ = 0.167] and an interaction Task-Set Transition and Session [*F*(1,30) = 4.7, *p* < 0. 05, η^2^ = 0.127] due to later P2 latency at t3 than t2 for non-switch (178 vs. 159 ms) and a shorter latency at t3 than t2 for task switch trials (155 vs. 162 ms). The P2 amplitude showed a large reduction after the combined REL + COG training (5.2 vs. 3.6 μV ms), resulting in a main effect of Session [*F*(1,30) = 54.5, *p* < 0.0001, η^2^ = 0.645]. No effect of Task-Set Transition or interaction Task-Set Transition × Session was found (Both *F*’s < 1). Finally, in order to assess possible effects of repeated measures comparison between t1 and t2 was conducted. ANOVA showed no significant effects or interactions either for P2 latency or amplitude.

In sum, the P2 amplitude was substantially reduced and the latency partly shortened both after the pure cognitive training (COG) and the combined relaxation and cognitive training (REL + COG). The P2 amplitude reduction remained stable during the follow-up measure.

#### N2

ANOVA analysing effects of the CT on the N2 latency showed an effect of Task-Set Transition [*F*(1,56) = 5.9, *p* < 0.05, η^2^ = 0.096], reflecting prolonged N2 latency in switch vs. non-switch trials (283 vs. 272 ms). There was a strong trend of Session [*F*(1,56) = 3.8, *p* = 0.054, η^2^ = 0.065] due to shorter latency at t2 than at t1 (172 vs. 184 ms). The interaction Session × Group did not reach significance [*F*(1,56) = 2.3, *p* = 0.13, η^2^ = 0.040].

In respect to N2 amplitude a main effect of Session indicated a larger (more negative) N2 at t2 than at t1 [-1.0 vs. -0.5 μV; *F*(1,56) = 6.2, *p* < 0.05, η^2^ = 0.100]. Most importantly, Session was modulated by Group [*F*(1,56) = 5.7, *p* < 0.05, η^2^ = 0.092], suggesting a N2 increase after COG training at t2 relative to t1 (-1.9 vs. -1.0 μV) while no difference in the Waiting Control Group was observed (-0.1 vs. -0.1 μV). The follow-up measure in the COG group showed no significant effects or interactions on N2 latency, whereas N2 amplitude was significantly reduced at t3 relative to t2 (-0.6 vs. -1.9 μV), resulting in an effect of Session [*F*(1,23) = 12.1, *p* < 0.005, η^2^ = 0.345]. Comparison between t1 and t3 did not yield any significant effects or interactions on N2 amplitude.

Assessment of the training effects in the REL + COG group showed an effect of Session on N2 latency [*F*(1,56) = 6.5, *p* < 0.05, η^2^ = 0.183], indicating a shorter N2 latency at t3 than at t2 (248 vs. 267 ms). No further effect or interaction reached significance. Comparison between t1 and t2 reflecting effects of repeated measure showed merely an effect of Task-Set Transition due to larger N2 in task switch than non-switch trials [-0.3 vs. 0.1 μV; *F*(1,56) = 5.8, *p* < 0.05, η^2^ = 0.157]. No effects of Session or interaction Session × Task-Set Transition was found.

In sum, N2 amplitude was increased after CT whereas no difference was found in the Waiting Control Group. However, the effect on N2 amplitude diminished in the follow-up measure. No significant training effect on N2 latency in the COG group was found. However, the N2 latency was shorter in the REL + COG group after training.

#### P3b

ANOVA did not show any effects or interactions on P3b latency. Analysis of P3b amplitude revealed an effect of Task-Set Transition, indicating smaller P3b in switch than non-switch trials [5.1 vs. 5.9 μV; *F*(1,56) = 12.8, *p* < 0.001, η^2^ = 0.187] and a trend of Session, showing a slight increase in P3b amplitude at t2 compared to t1 [6.2 vs. 6.8 μV; *F*(1,56) = 3.5, *p* = 0.06, η^2^ = 0.059]. There was no interaction with Group (*F*’s < 1). However, the main effect of Group showed a larger P3b in the control than in the training group [6.5 vs. 4.5 μV; *F*(1,56) = 6.5, *p* < 0.05, η^2^ = 0.105]. The follow-up measure showed no substantial changes of the P3b between t2 and t3.

The ANOVA analysing the effects of the combined training (REL + COG) yielded an effect of Session, indicating P3b reduction from t2 to t3 [6.9 vs. 5.8 μV; *F*(1,30) = 7.5, *p* < 0.01, η^2^ = 0.201]. No effects on P3b latency were found. Finally, the comparison t1 vs. t2 in this group showed apart from the standard effect of Task-Set Transition [6.9 vs. 6.0 μV; *F*(1,31) = 8.8, *p* < 0.01, η^2^ = 0.222] a trend of Session, suggesting P3b increase from t1 to t2 [6.2 vs. 6.8 μV; *F*(1,31) = 3.7, *p* = 0.06, η^2^ = 0.108].

Taken together, the P3b amplitude and latency did not vary consistently as a function of training. Its amplitude increased at t2 and decreased at t3. The Waiting Control Group showed generally larger P3b amplitude than the COG group.

#### Nc and Ne

**FIGURE 6:**
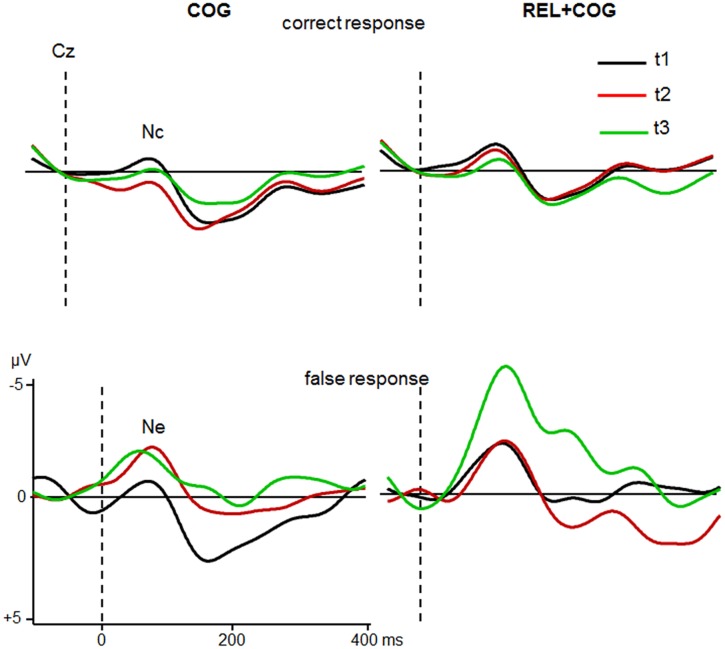
**Nc and Ne at Cz of the COG and the Waiting Control Group (REL + COG) for correct (Top)** and false response **(Bottom)** as a function Sessions t1 (black), t2 (red), and t3 (green). See caption of **Figure 3** and the study design for more information.

The correct response negativity (Nc) and error negativity (Ne) at Cz (**Figure 6**) were pooled across switch and non-switch trials. There were 24 participants in each group that made a sufficient number of erroneous responses. There were no effects or interactions on Nc or Ne latency.

The ANOVA including the factors Response Correctness (Nc, Ne), Session and Group showed an effect of Correctness [*F*(1,46) = 26.8, *p* < 0.0001, η^2^ = 0.368] with larger amplitudes in error than correct trials (-3.1 vs. -1.0 μV), an interaction Correctness and Session [*F*(1,46) = 6.6, *p* < 0.05, η^2^ = 0.125] due to larger increase in the Ne (-2.6 vs. -3.6 μV) than Nc (-1.3 vs. -0.7 μV) between t1 and t2. Most importantly, the interaction of all three factors was also significant [*F*(1,46) = 4.6, *p* < 0.05, η^2^ = 0.091], indicating a larger increase in the Ne after the CT whereas no difference was found for the Control Group. Comparison between t1 and t2 for Ne in the COG group confirmed this result [-1.8 vs. -3.6 μV; *F*(1,23) = 4.3, *p* < 0.05, η^2^ = 0.158], whereas no difference between t1 and t2 was found in the Waiting Control Group (-3.4 vs. -3.6 μV; *F* < 1). The Ne amplitude in the COG group remained at the same level in the follow-up measure (-3.7 μV; *F* < 1). An additional comparison between t1 and t3 revealed a trend [-1.8 vs. -3.6 μV; *F*(1,20) = 3.5, *p* = 0.07, η^2^ = 0.150].

For the combined REL + COG group, the ANOVA showed again an effect of Correctness due to larger amplitude in error than correct trials [-5.6 vs. -1.2 μV; *F*(1,18) = 19.5, *p* < 0.0001, η^2^ = 0.520], an effect of Session, suggesting an amplitude increase between t2 and t3 [-2.6 vs. -4.2 μV; *F*(1,18) = 4.8, *p* < 0.05, η^2^ = 0.210] and an interaction Correctness × Session [*F*(1,18) = 7.1, *p* < 0.05, η^2^ = 0.284], showing a strong increase in the Ne between t2 and t3 [-3.8 vs. -7.3 μV; *F*(1,18) = 6.3, *p* < 0.05, η^2^ = 0.259], while the Nc showed similar amplitudes at t2 and t3 (-1.4 vs. -1.0). The comparison between t1 and t3 corroborated the training-related increase in the Ne [*F*(1,18) = 15.4, *p* < 0.001, η^2^ = 0.448], which cannot be attributed to repeated measures as indicated by the non-significant comparison between t1 and t2 (*F* < 1).

In sum, the Ne after erroneous responses was, as expected, larger than the Nc after correct responses. The Ne (but not the Nc) was larger after the CT and remained stable during the follow-up measure while no changes were found in the Waiting Control Group. However, after the combined training (REL + COG) this group also showed a strong enhancement of the Ne.

## Discussion

The aim of the present study was the enhancement of executive control processes in middle-aged industry employees with unchallenging, repetitive type of work by a 3 months, trainer- guided CT or a combined relaxation/stress management and cognitive intervention. Pre- and post-measure in the Cognitive Training and the Waiting Control Group allowed assessing the effects of the CT. A follow-up measure was included to assess the residual effects of training 3 months after the training was finished. The Waiting Control Group received a combined relaxation and CT (REL + COG) lasting also 3 months after the post-measure was completed. The effects of the combined training were evaluated after the training was finished.

Training effects were assessed using a complex memory based task switching paradigm which was shown to be sensitive to a number of cognitive or physical interventions (Gajewski and Falkenstein, 2012, 2015a). In accordance with the previous study with seniors (Gajewski and Falkenstein, 2012) CT substantially improved the accuracy in this task, whereas RTs were not affected. The new finding of the present study is that this improvement remained stable 3 months after the training ended. An additional support for this finding provided the Waiting Control Group showing a similar improvement in accuracy after the REL + COG training. However, one point may be problematic for the interpretation of training effects in the present study. At first glance the different ERs at t1 between the groups may question the real training effect as the chance to reduce ERs was rather higher in the COG group due to poorer initial performance than in the REL + COG group (despite randomization procedure). However, the statistical analysis showed unequivocally significant improvement in accuracy both in the COG group as well as in the REL +COG group. In other words, the REL + COG group did not reduce ERs between t1 and t2 but reduced it significantly after training, indicating no effects of repeated measures and a real effect of training even if the initial accuracy was high.

Electrophysiological data allowed further insights in the specific processing steps and cognitive subcomponents influenced by training. The target-locked ERPs provide a number of interesting findings concerning effects of the CT. First, the amplitude of the P2 was consistently reduced and its latency shortened both after CT alone and after combined training. This attenuation remained stable during the follow-up measure and was not caused by repeated measures. Its reduced amplitude and latency after training may reflect less effort during task-set retrieval that leads to a faster activation of task relevant stimulus-response sets (c.f. Finke et al., 2011; Schapkin et al., 2014).

The next process which was influenced by training in the present study was the frontocentral N2. Our previous study with seniors already showed the sensitivity of the N2 to CT (Gajewski and Falkenstein, 2012). Apart from the usually observed effects of a larger N2 in task switch than non-switch trials, the N2 amplitude was increased both after the pure CT and the combined cognitive and relaxation training. The enhanced N2 suggests a more intense response selection and may be the origin of the improved accuracy. No training effects on N2 latency were found. A surprising finding in this study was the reduction in the amplitude enhancement after a 3-month training free period. This may suggest either an adaptation effect of neuronal structures underlying response selection or alternatively an index of gradual decay of the training induced effect. Both possibilities should be considered in future studies.

The P3b showed a lower amplitude in switch vs. non-switch trials, which was consistently reported previously (Barceló et al., 2000; Rushworth et al., 2002; Jost et al., 2008; Gajewski and Falkenstein, 2011, 2012, 2015a; Karayanidis et al., 2011). However, the P3b did not vary as a function of training. Its amplitude was generally increased during pre-measure and reduced during follow-up measure but this effect was probably not related to the training but rather due to the repeated application of the same task and corresponding adaptation effects. As the P3b is a conglomerate of diverse neuronal mechanisms (e.g., Polich, 2007) a simpler paradigm like Oddball paradigm may be more appropriate to analyze P3b changes in the course of CT.

Finally, the most consistent results associated with training was provided by the analysis of error negativity Ne. The Ne was expectedly more pronounced than the corresponding negativity during correct responses. Most importantly, the Ne (but not the Nc) was substantially increased after CT in both groups (COG, COG + REL) and remained stable during the follow-up measure for the COG group while no changes were found in the Waiting Control Group. These results corroborate findings obtained in our previous study with seniors (Gajewski and Falkenstein, 2012). The improvement of accuracy was previously interpreted as a consequence of the enhancement of the response selection process reflected by the N2. This may lead to higher awareness about the correct response which in turn produces a strong signal if the expectation to respond correctly was violated.

The present study extended previous results obtained with middle-aged employees of the same big car factory that showed cognitive impairments in assembly line workers compared to flexibly working employees responsible for service, maintenance and repair of machines (Gajewski et al., 2010b). The assembly line employees showed increased ERs and RTs in the same task as in the present study and reduced P3b and Ne relative to their flexible working colleagues. In the present study, the initial cognitive status of the participants was comparable to the performance of those of the previous study as the participants were derived from the same population of middle-aged assembly line workers. The training effects obtained in the present study indicate that 3 months of cognitive or combined relaxation training is able to reduce cognitive decline and to elevate cognitive performance to the level of flexibly working employees.

Unfortunately, these findings do not allow drawing conclusions regarding the employability of the elderly workers as no far transfer tasks related to the work were used. However, it is plausible to assume that the observed gains of cognitive functions may improve the ability to learn new contents and work processes, enhance self-confidence and help to strengthen individual potential at work. Therefore, future studies in occupational environments should also evaluate far transfer by measurements of work efficiency, individual performance at work, risk of workplace injuries and work-related illness.

In summary, CT appears a most promising tool for improving mental fitness and employability in older workers. Future CT studies in occupational environments should evaluate far transfer by measurements of work efficiency, individual performance at work, risk of workplace injuries and work-related illness.

### Limitations

The present study has a number of limitations which have to be acknowledged. Firstly, much effort has been invested to acquire the sample, as a large number of industry workers of this car factory were not willing to participate in the relatively time-consuming study before or after their work. Therefore, we assume that the study population was not fully representative of the factory population. Secondly, the age of the participants was rather homogeneous (mid forty). It would be interesting to analyze training effects in older employees, beyond sixties as older workers may show larger cognitive deficits which may be restored to improve or maintain their quality of life and employability until their retirement. Thirdly, the experimental design was not fully balanced and the training effects in the COG and REL + COG Group not directly comparable. Fourthly, the effects of CT were not specific to the switch ability as no differential effects in non-switch and switch trials were found. Reduction of local switch costs would be indicative for improvements in the switch ability, whereas performance enhancement in non-switch trials would be indicative for improvements in mixing costs. Indeed, mixing costs are a more sensitive parameter in respect to physical or CT (Gajewski and Falkenstein, 2012, 2015a). In other words, it is quite possible that other functions were improved by the training as a wide range of cognitive abilities was trained. Our assumption is that CT does not affect the switch ability *per se* but rather enhance updating of relevant task information and working memory capacity that improve the ability to maintain a long task sequence as in the current paradigm and to activate the relevant task-set at the right time point. This was supported by the observation that the errors in this paradigm are mainly due to lapses in the representation of the task sequence and activating the wrong task-set. Finally, and related to the previous point, the CT was qualitatively heterogeneous and trained a large number of cognitive functions. Therefore, it is not possible to determine which specific training regime was effective to affect the data of our study and which trainings were fully ineffective. Nevertheless, as this study was conducted in an applied context, controlling of a large number of confounding factors was not possible. Future studies with more controlled training regimes should be conducted to establish the most efficient training conditions in respect to duration, intensity, and content.

## Conclusion

The present study was designed to compensate cognitive deficits in middle-aged workers with unchallenging work. Training-related gains in cognitive performance were observed in an improved accuracy, suggesting enhanced maintenance of a complex task sequence in working memory. This performance benefit was accompanied by a number of electrophysiological effects, like amplitude decrease and latency reduction of a frontocentral P2 related to retrieval of task-sets and latency decrease and amplitude increase of the frontocentral N2 associated with selection of a correct response. The P2 effect persisted 3 months after the training was finished, whereas the N2 amplitude effect disappeared.

Furthermore, an increase in error negativity (Ne) associated with error detection and monitoring was found which also persisted after a 3 months training-free period. In contrast, no performance and ERP effects in the Control Group were observed. Though, after the Waiting Control Group received the combined cognitive and relaxation training, the same performance and ERP changes were observed as in the Cognitive Training Group, suggesting that these effects are unequivocally due to training and not to the effect of repeated measures. These findings suggest that formal CT may indeed ameliorate cognitive decline that is also reflected in a sustained improvement of brain activity.

## Author Contributions

PG: Conception and design, data analysis, interpretation of the data, writing, and final approval. GF and MF: Conception and design, interpretation of the data, writing, and final approval.

## Conflict of Interest Statement

The authors declare that the research was conducted in the absence of any commercial or financial relationships that could be construed as a potential conflict of interest.

## Footnotes

1 www.brain-trainer.de

2 www.happyneuron.de

3 www.ahano.de

4 www.freshminder.de

5 www.mentaga.de
